# Supplementary material for: A Meta-Omics Analysis Unveils the Shift in Microbial Community Structures and Metabolomics Profiles in Mangrove Sediments Treated with a Selective Actinobacterial Isolation Procedure
Source: Molecules. 2021 Dec 2;26(23):7332. doi: 10.3390/molecules26237332 (PMC8658942; doi:10.3390/molecules26237332)
Supplement: Supplementary file 1 [file molecules-26-07332-s001.zip › molecules-1431585-supplementary.pdf]

molecules

MDPI

Supplementary Materials

A Meta-Omics Analysis Unveils the Shift in Microbial Community Structures and Metabolomics Profiles in Mangrove Sediments Treated with a Selective Actinobacterial Isolation Procedure

Miguel David Marfil-Santana [1], Anahí Martínez-Cárdenas [2], Analuisa Ruíz-Hernández [1], Mario Vidal-Torres [1], Norma Angélica Márquez-Velázquez [1], Mario Figueroa [2] and Alejandra Prieto-Davó [1,*]

[1] Unidad de Química-Sisal, Facultad de Química, Universidad Nacional Autónoma de México, Sisal 97356, Mexico; miguelmarfil81@gmail.com (M.D.M.-S.); aluisarh@hotmail.com (A.R.-H.); mario-fate@hotmail.com (M.V.-T.); angelica.marvel@quimica.unam.mx (N.A.M.-V.)
[2] Facultad de Química, Universidad Nacional Autónoma de México, Ciudad de México 04510, Mexico; amartinez@quimica.unam.mx (A.M.-C.); mafiguer@unam.mx (M.F.)
* Correspondence: apdavo@unam.mx

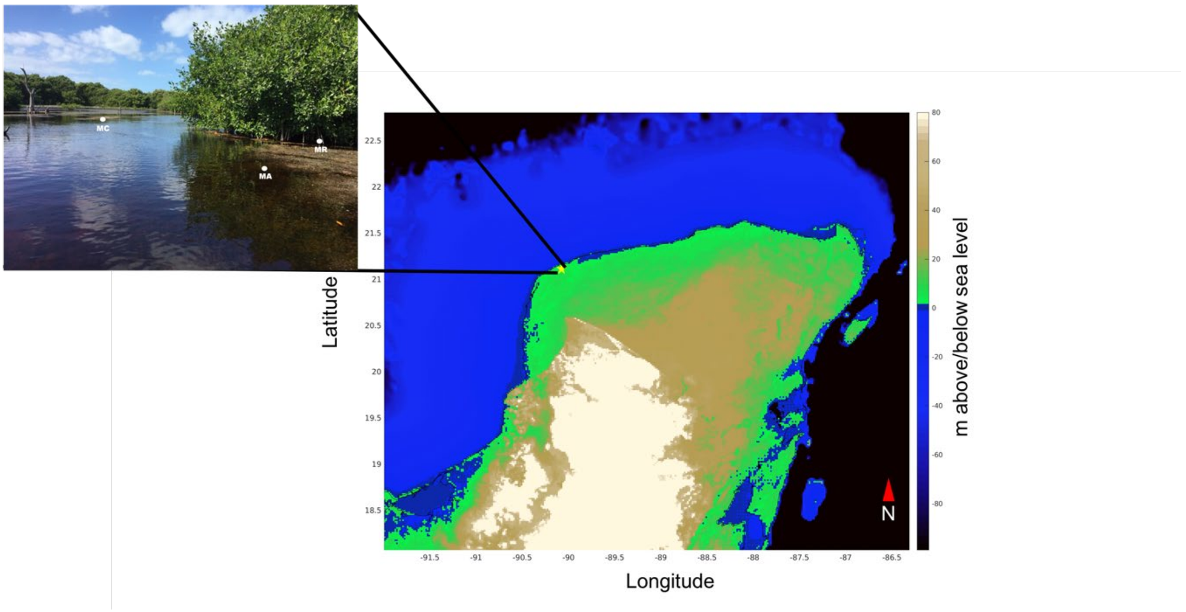

Figure S1. Samples obtained from mangrove sediments at Sisal Yucatán. MR: sediments collected above mangrove roots. MA: sediments collected c.a. 1.5 m from mangrove roots. MC: sediments collected from running water channel with no mangrove root influence. T0: sediments from mangrove water channel (MC) sampled in 2018. TA: enrichment water control. TM and TMa: enrichment experiments without and with antibiotics (cycloheximide 10 µg/ml, gentamycin 10 µg/ml and rifampicin 1 µg/ml), respectively.

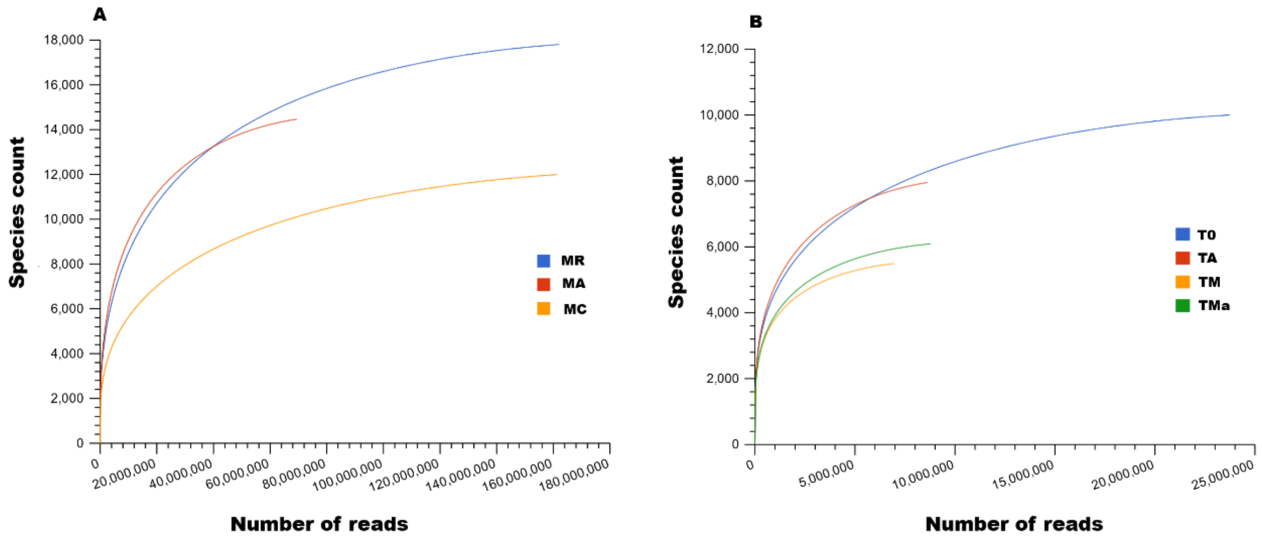

Figure S2. Rarefaction curves for sequenced reads show that saturation was reached with all samples analyzed. **A**) Sequences from mangrove sediments sampled in 2017. **B**) Sequences from mangrove sediments (MC location only) sampled in 2018 (T0) and enrichment experiments (TM, TMa) with autoclaved mangrove water control (TA).

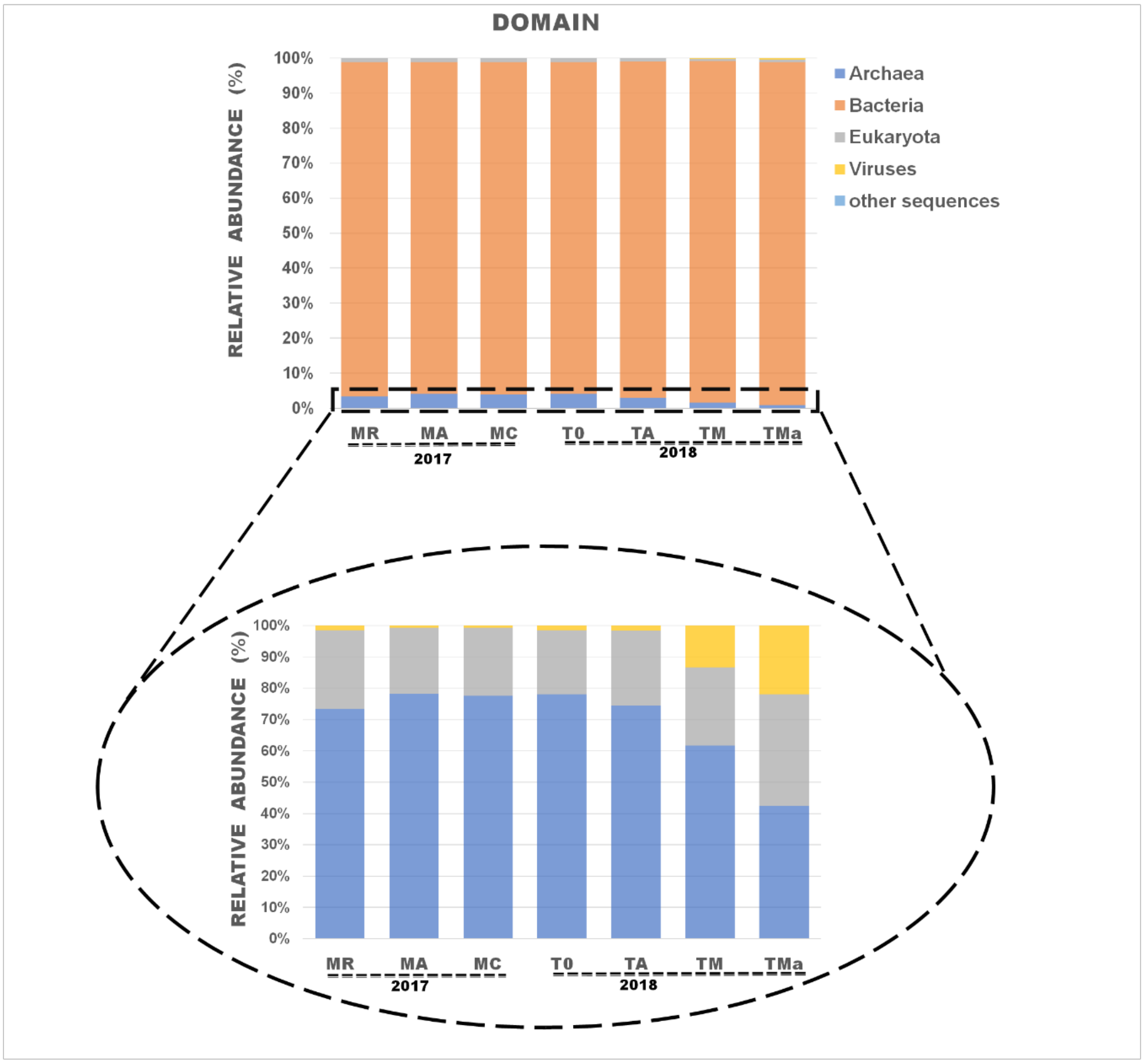

Figure S3. Relative abundance at the Domain level for all metagenomes analyzed. MR: sediments collected above mangrove roots. MA: sediments collected c.a. 1.5 m from mangrove roots. MC: sediments collected from running water channel with no mangrove root influence. T0: sediments from mangrove water channel (MC) sampled in 2018. TA: enrichment water control. TM and TMa: enrichment experiments without and with antibiotics (cycloheximide 10 µg/ml, gentamycin 10 µg/ml and rifampicin 1 µg/ml), respectively.

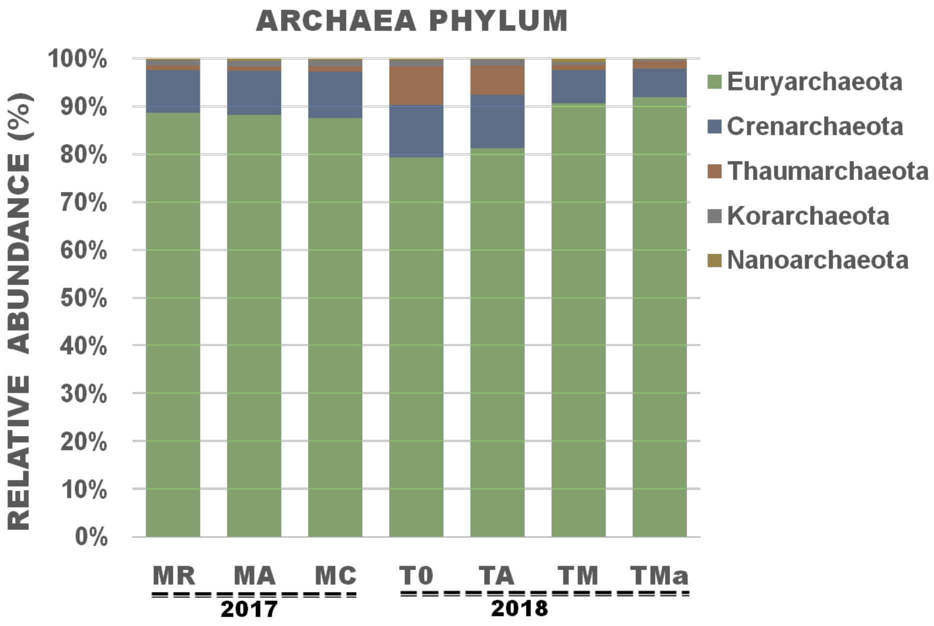

Figure S4. Relative abundance of the phylum Archaea. MR: sediments collected above mangrove roots. MA: sediments collected c.a. 1.5 m from mangrove roots. MC: sediments collected from running water channel with no mangrove root influence. T0: sediments from mangrove water channel (MC) sampled in 2018. TA: enrichment water control. TM and TMa: enrichment experiments without and with antibiotics (cycloheximide 10 μg/ml, gentamycin 10 μg/ml and rifampicin 1 μg/ml), respectively.

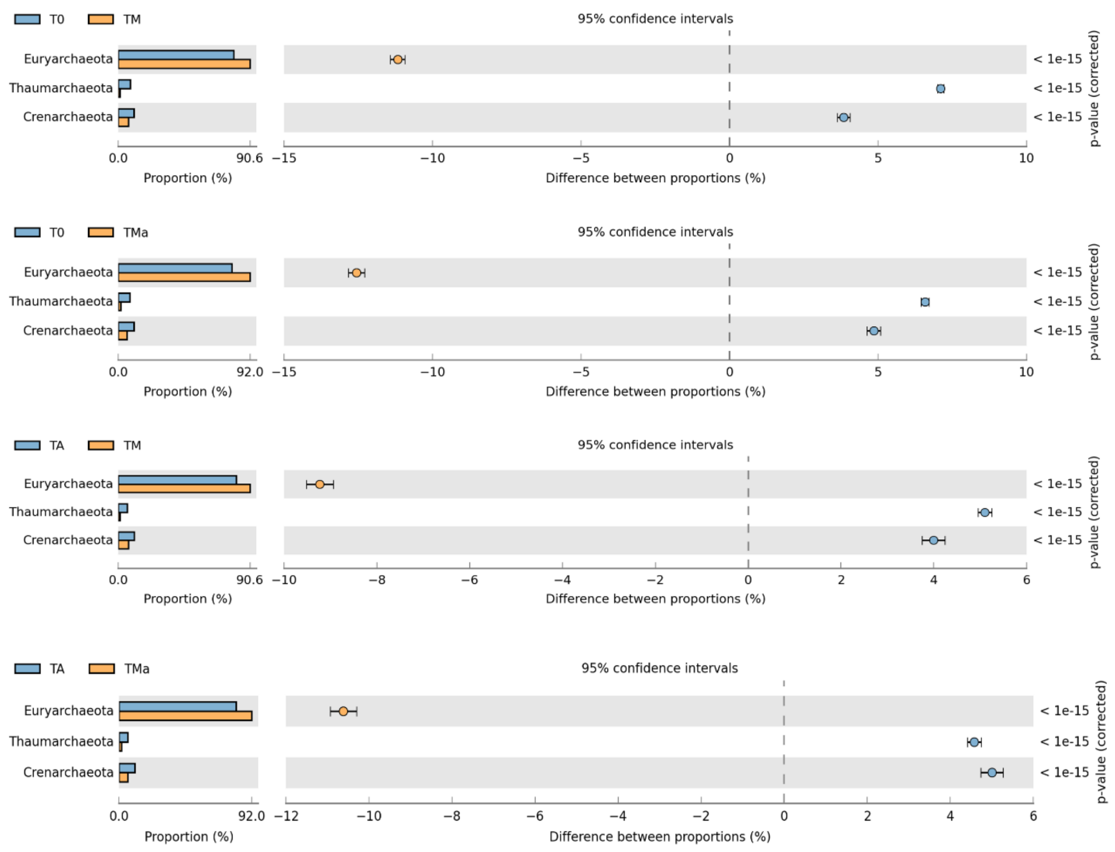

Figure S5. Statistically significant ($p < 0.05$) differences observed between archaeal phyla abundances from the metagenomes analyzed using STAMP. MR: sediments collected above mangrove roots. MA: sediments collected c.a. 1.5 m from mangrove roots. MC: sediments collected from running water channel with no mangrove root influence. T0: sediments from mangrove water channel (MC) sampled in 2018. TA: enrichment water control. TM and TMa: enrichment experiments without and with antibiotics (cycloheximide 10 µg/ml, gentamycin 10 µg/ml and rifampicin 1 µg/ml), respectively.

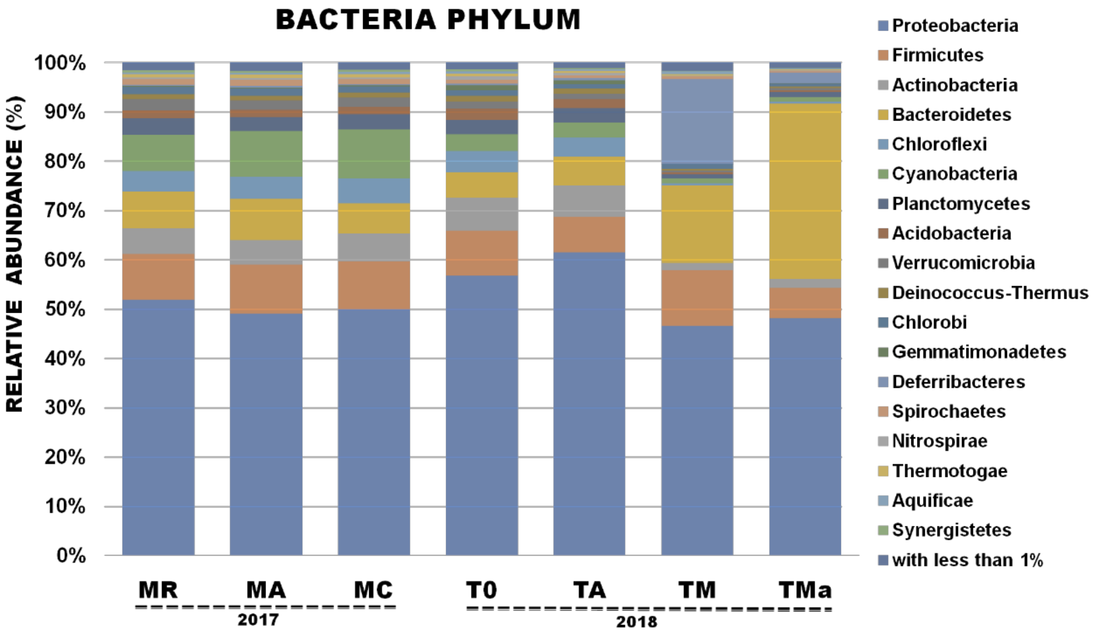

Figure S6. Relative abundance of bacterial phyla for all metagenomes analyzed. MR: sediments collected above mangrove roots. MA: sediments collected c.a. 1.5 m from mangrove roots. MC: sediments collected from running water channel with no mangrove root influence. T0: sediments from mangrove water channel (MC) sampled in 2018. TA: enrichment water control. TM and TMa: enrichment experiments without and with antibiotics (cycloheximide 10 µg/ml, gentamycin 10 µg/ml and rifampicin 1 µg/ml), respectively.

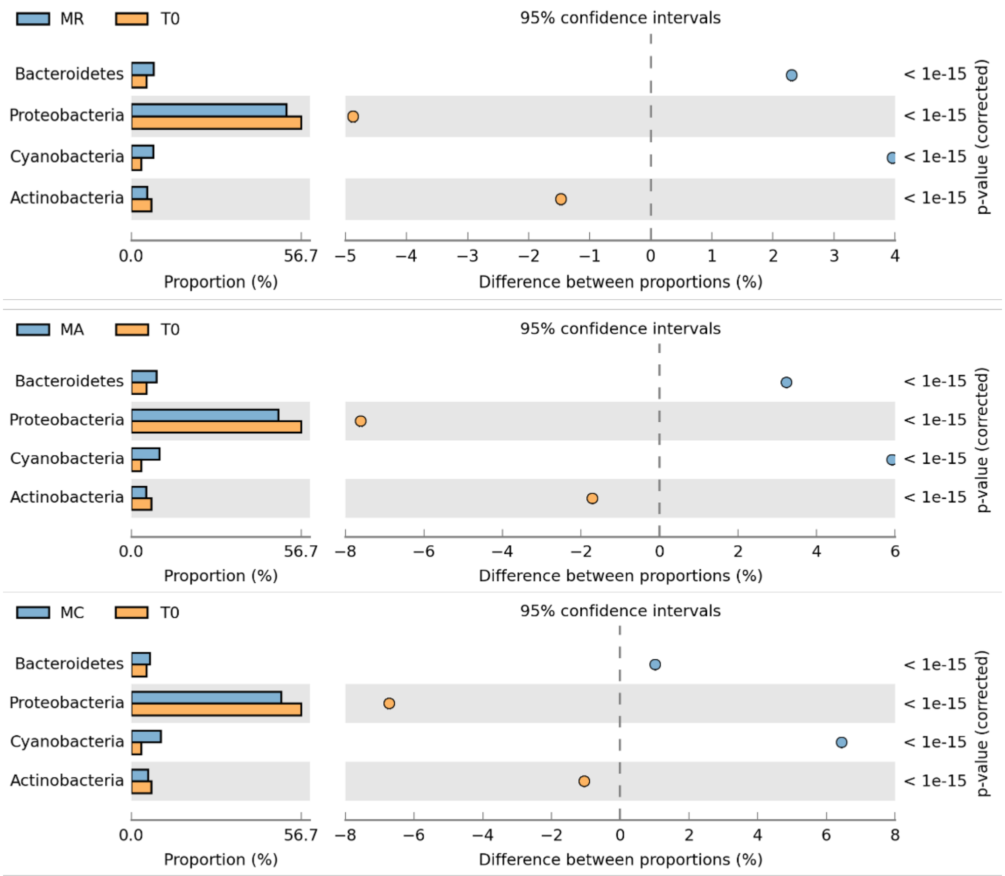

Figure S7. Statistically significant ($p < 0.05$) differences among bacterial phyla abundances from the metagenomes analyzed using STAMP. MR: sediments collected above mangrove roots. MA: sediments collected c.a. 1.5 m from mangrove roots. MC: sediments collected from running water channel with no mangrove root influence. T0: sediments from mangrove water channel (MC) sampled in 2018. TA: enrichment water control. TM and TMa: enrichment experiments without and with antibiotics (cycloheximide 10 µg/ml, gentamycin 10 µg/ml and rifampicin 1 µg/ml), respectively.

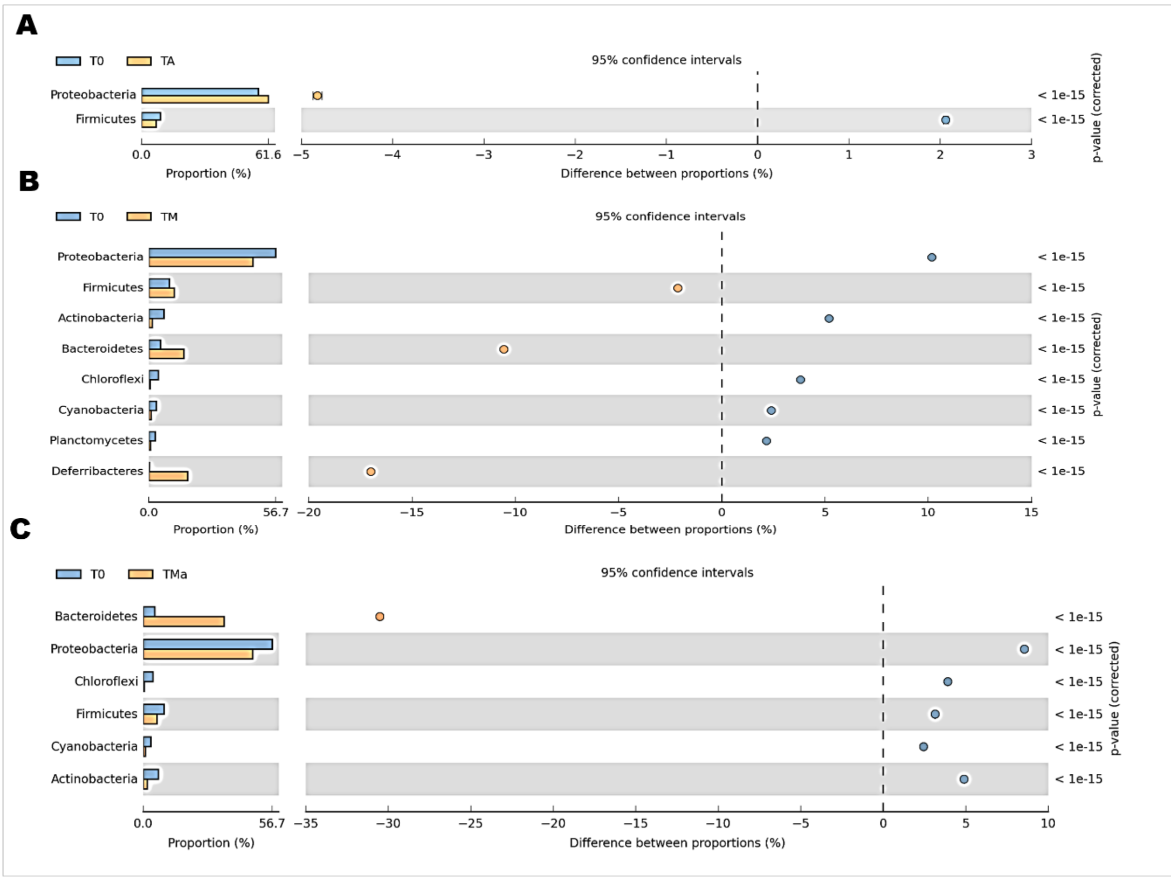

Figure S8. Statistically significant ($p < 0.05$) differences among bacterial phyla abundances from the metagenomes analyzed using STAMP. MR: sediments collected above mangrove roots. MA: sediments collected c.a. 1.5 m from mangrove roots. MC: sediments collected from running water channel with no mangrove root influence. T0: sediments from mangrove water channel (MC) sampled in 2018. TA: enrichment water control. TM and TMa: enrichment experiments without and with antibiotics (cycloheximide 10 µg/ml, gentamycin 10 µg/ml and rifampicin 1 µg/ml), respectively.

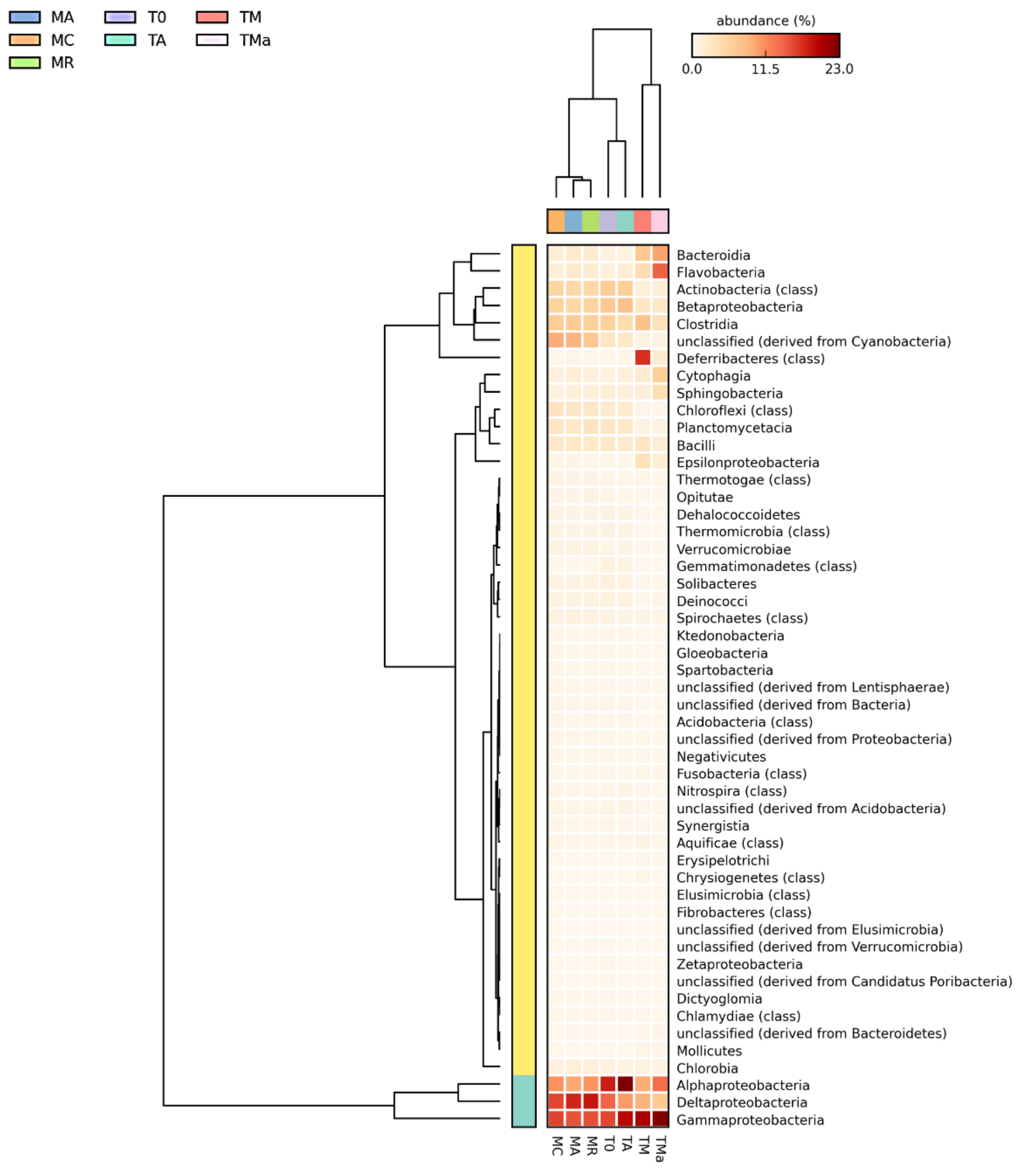

Figure S9. Heat map for bacterial Class abundance. MR: sediments collected above mangrove roots. MA: sediments collected c.a. 1.5 m from mangrove roots. MC: sediments collected from running water channel with no mangrove root influence. T0: sediments from mangrove water channel (MC) sampled in 2018. TA: enrichment water control. TM and TMa: enrichment experiments without and with antibiotics (cycloheximide 10 µg/ml, gentamycin 10 µg/ml and rifampicin 1 µg/ml), respectively.

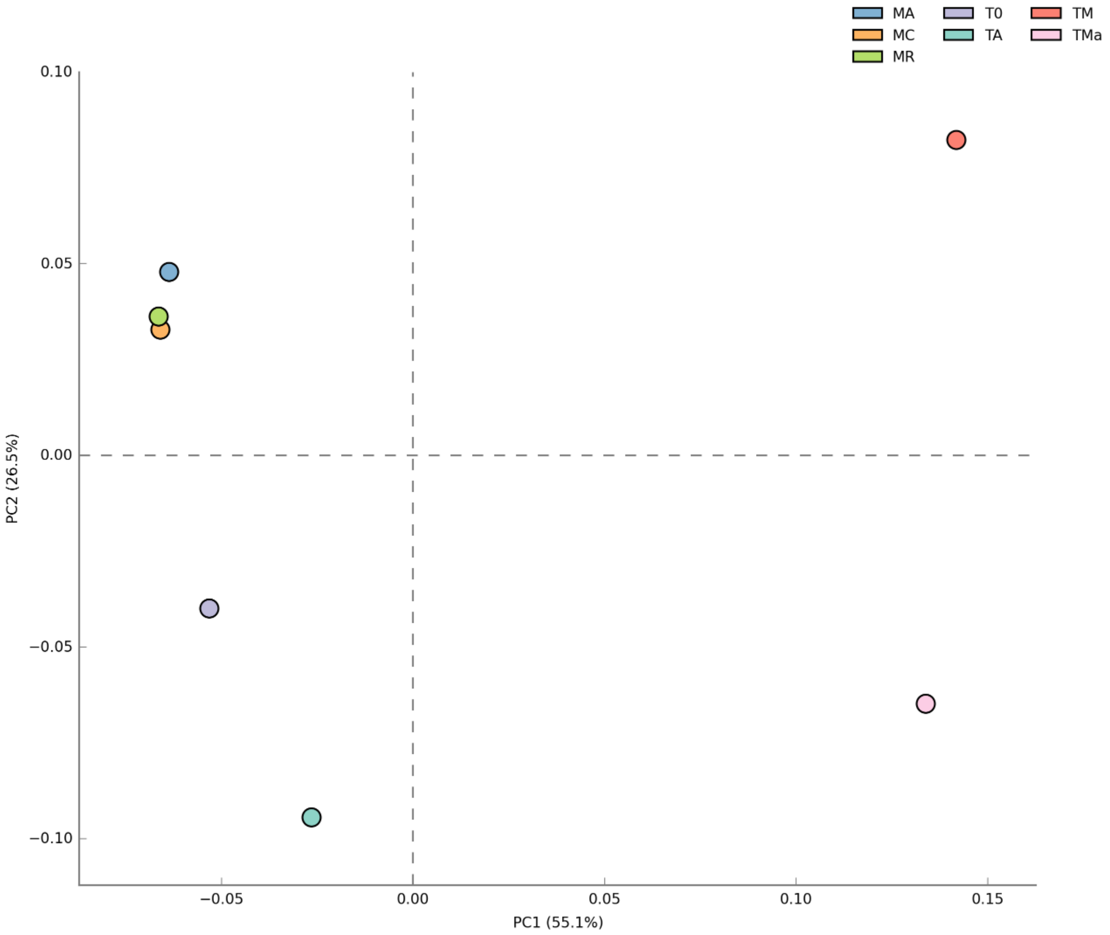

Figure S10. Principal component analysis (PCA) of Bacteria at the Order taxonomic level for all metagenomes studied. MR: sediments collected above mangrove roots. MA: sediments collected c.a. 1.5 m from mangrove roots. MC: sediments collected from running water channel with no mangrove root influence. T0: sediments from mangrove water channel (MC) sampled in 2018. TA: enrichment water control. TM and TMa: enrichment experiments without and with antibiotics (cycloheximide 10 µg/ml, gentamycin 10 µg/ml and rifampicin 1 µg/ml), respectively.

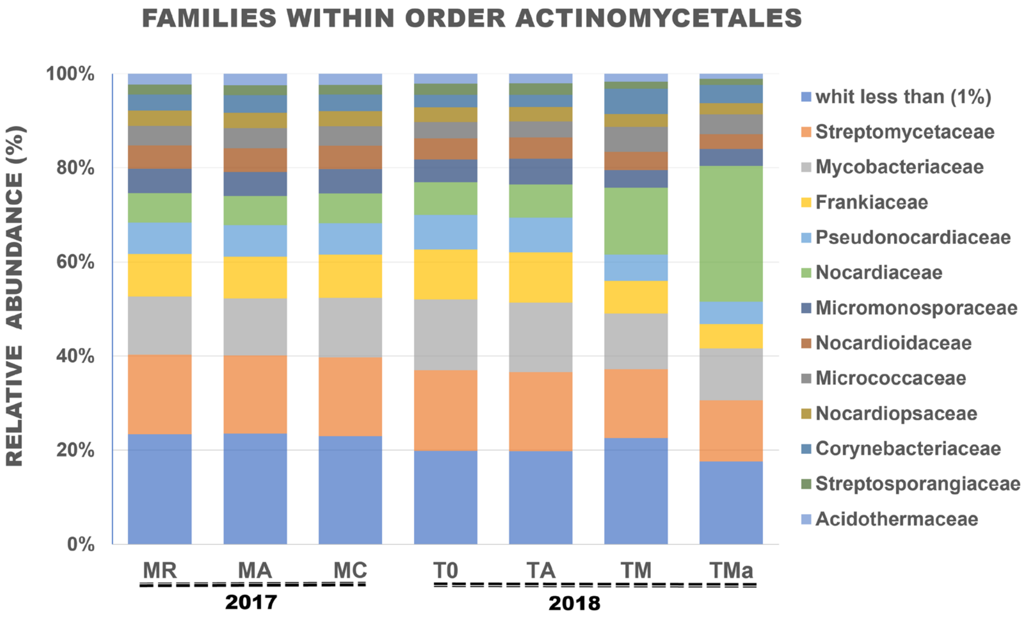

Figure S11. Relative abundance of families within the Order Actinomycetales (Class Actinomycetia) from all metagenomes analyzed. MR: sediments collected above mangrove roots. MA: sediments collected c.a. 1.5 m from mangrove roots. MC: sediments collected from running water channel with no mangrove root influence. T0: sediments from mangrove water channel (MC) sampled in 2018. TA: enrichment water control. TM and TMa: enrichment experiments without and with antibiotics (cycloheximide 10 µg/ml, gentamycin 10 µg/ml and rifampicin 1 µg/ml), respectively.

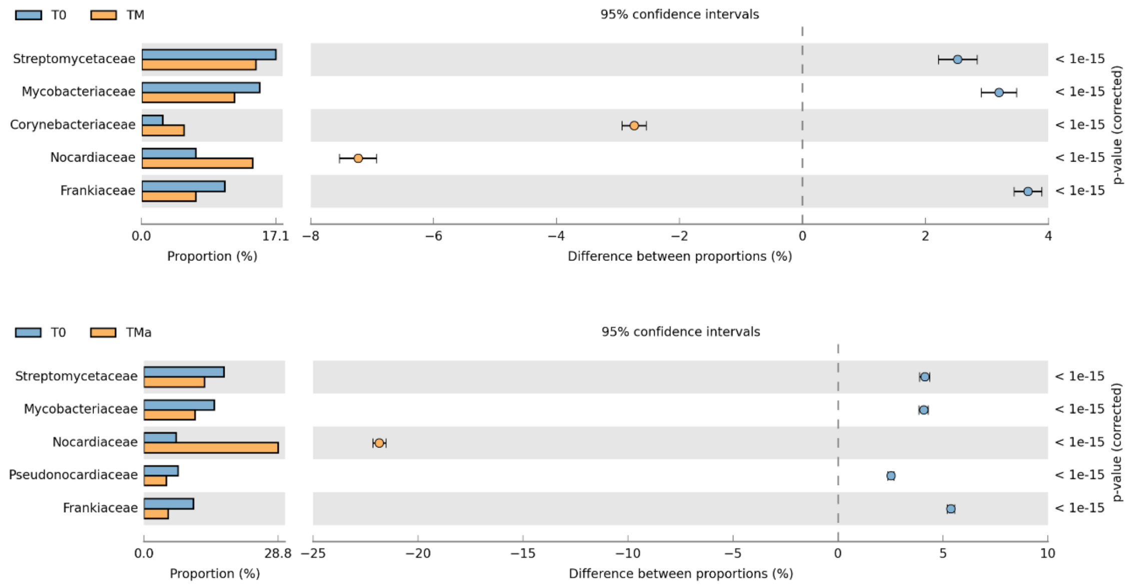

Figure S12. Statistically significant differences ($p < 0.05$) within the families in the Order Actinomycetales for all metagenomes analyzed by STAMP. MR: sediments collected above mangrove roots. MA: sediments collected c.a. 1.5 m from mangrove roots. MC: sediments collected from running water channel with no mangrove root influence. T0: sediments from mangrove water channel (MC) sampled in 2018. TA: enrichment water control. TM and TMa: enrichment experiments without and with antibiotics (cycloheximide 10 µg/ml, gentamycin 10 µg/ml and rifampicin 1 µg/ml), respectively.

.

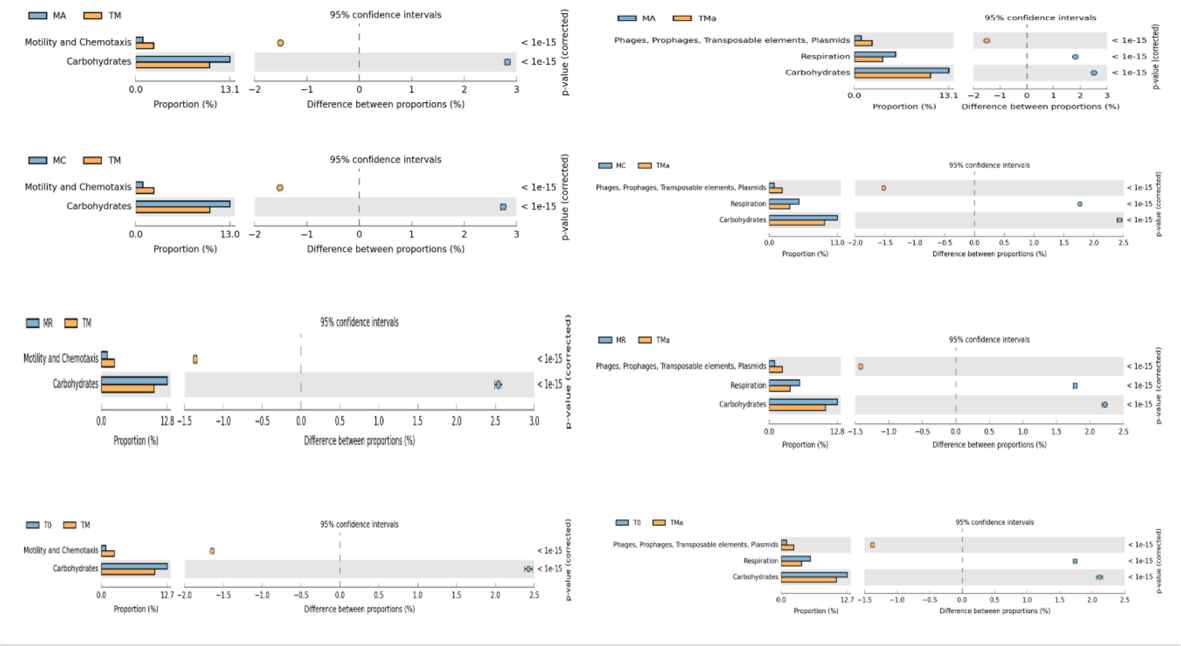

Figure S13. Statistically significant differences ($p < 0.05$) among the first level (MG-RAST) metabolisms among the studied metagenomes by STAMP. MR: sediments collected above mangrove roots. MA: sediments collected c.a. 1.5 m from mangrove roots. MC: sediments collected from running water channel with no mangrove root influence. T0: sediments from mangrove water channel (MC) sampled in 2018. TA: enrichment water control. TM and TMa: enrichment experiments without and with antibiotics (cycloheximide 10 µg/ml, gentamycin 10 µg/ml and rifampicin 1 µg/ml), respectively.

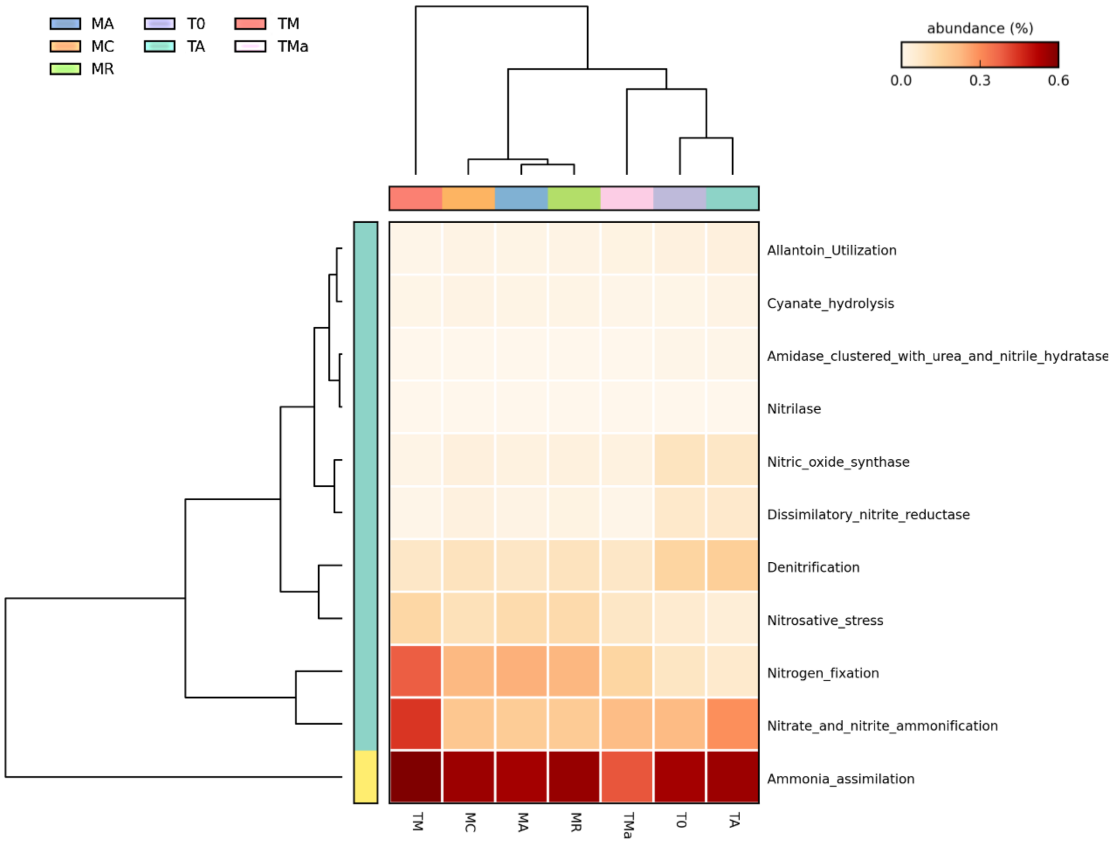

Figure S14. Heat map for nitrogen metabolism genes (MG-RAST) in all metagenomes analyzed. MR: sediments collected above mangrove roots. MA: sediments collected c.a. 1.5 m from mangrove roots. MC: sediments collected from running water channel with no mangrove root influence. T0: sediments from mangrove water channel (MC) sampled in 2018. TA: enrichment water control. TM and TMa: enrichment experiments without and with antibiotics (cycloheximide 10 µg/ml, gentamycin 10 µg/ml and rifampicin 1 µg/ml), respectively.

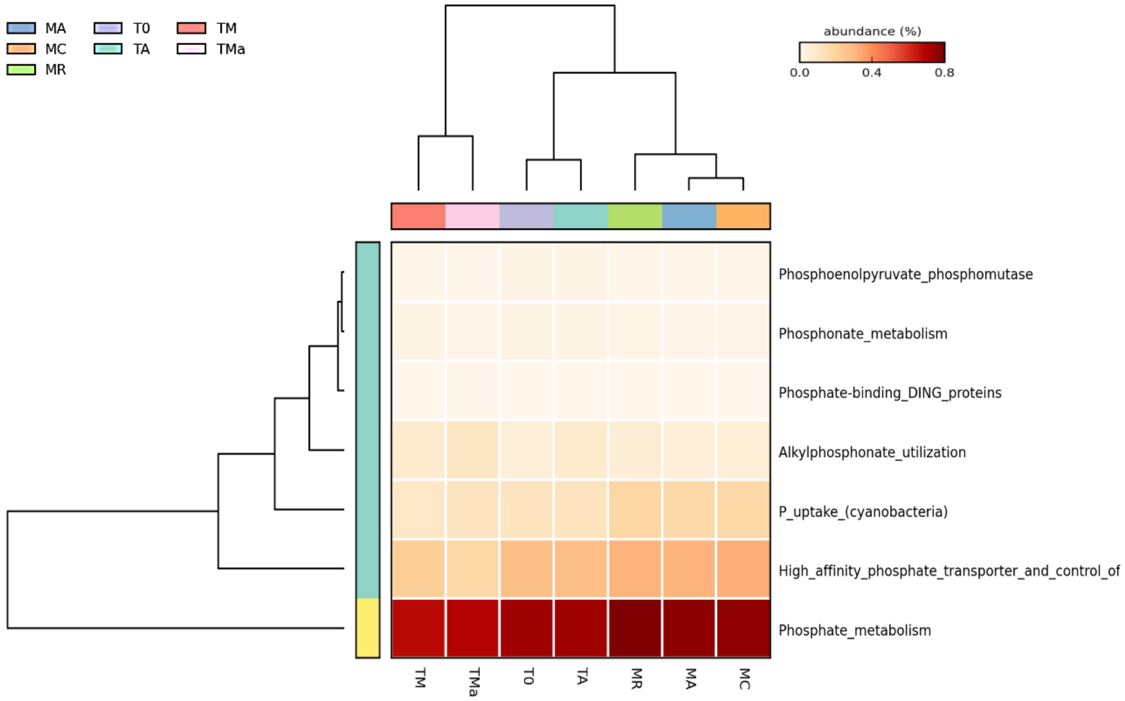

Figure S15. Heat map for phosphorous metabolism in all metagenomes analyzed. MR: sediments collected on top of mangrove roots. MA: sediments collected 1.5 m from mangrove roots. MC: sediments from running water channel with no mangrove root influence. T0: mangrove water channel (MC) sampled in 2018. TA: experiment control. TM and TMa: enrichment experiments without and with antibiotics (cycloheximide 10 µg/ml, gentamycin 10 µg/mL and rifampicin 1 µg/mL), respectively.

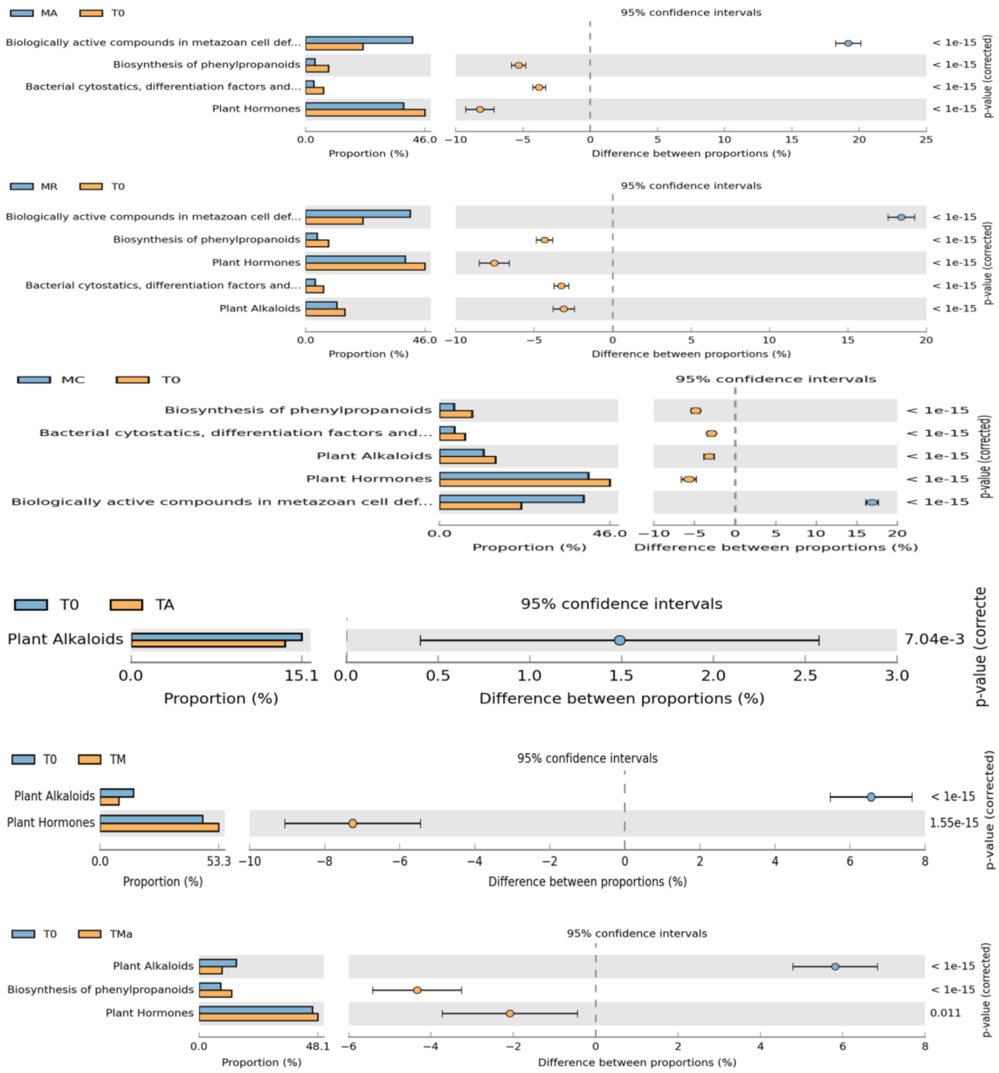

Figure S16. Significant differences ($p < 0.05$) in secondary metabolism level 3 (MG-RAST) among analyzed metagenomes by STAMP. MR: sediments collected above mangrove roots. MA: sediments collected c.a. 1.5 m from mangrove roots. MC: sediments collected from running water channel with no mangrove root influence. T0: sediments from mangrove water channel (MC) sampled in 2018. TA: enrichment water control. TM and TMa: enrichment experiments without and with antibiotics (cycloheximide 10 µg/ml, gentamycin 10 µg/ml and rifampicin 1 µg/ml), respectively.

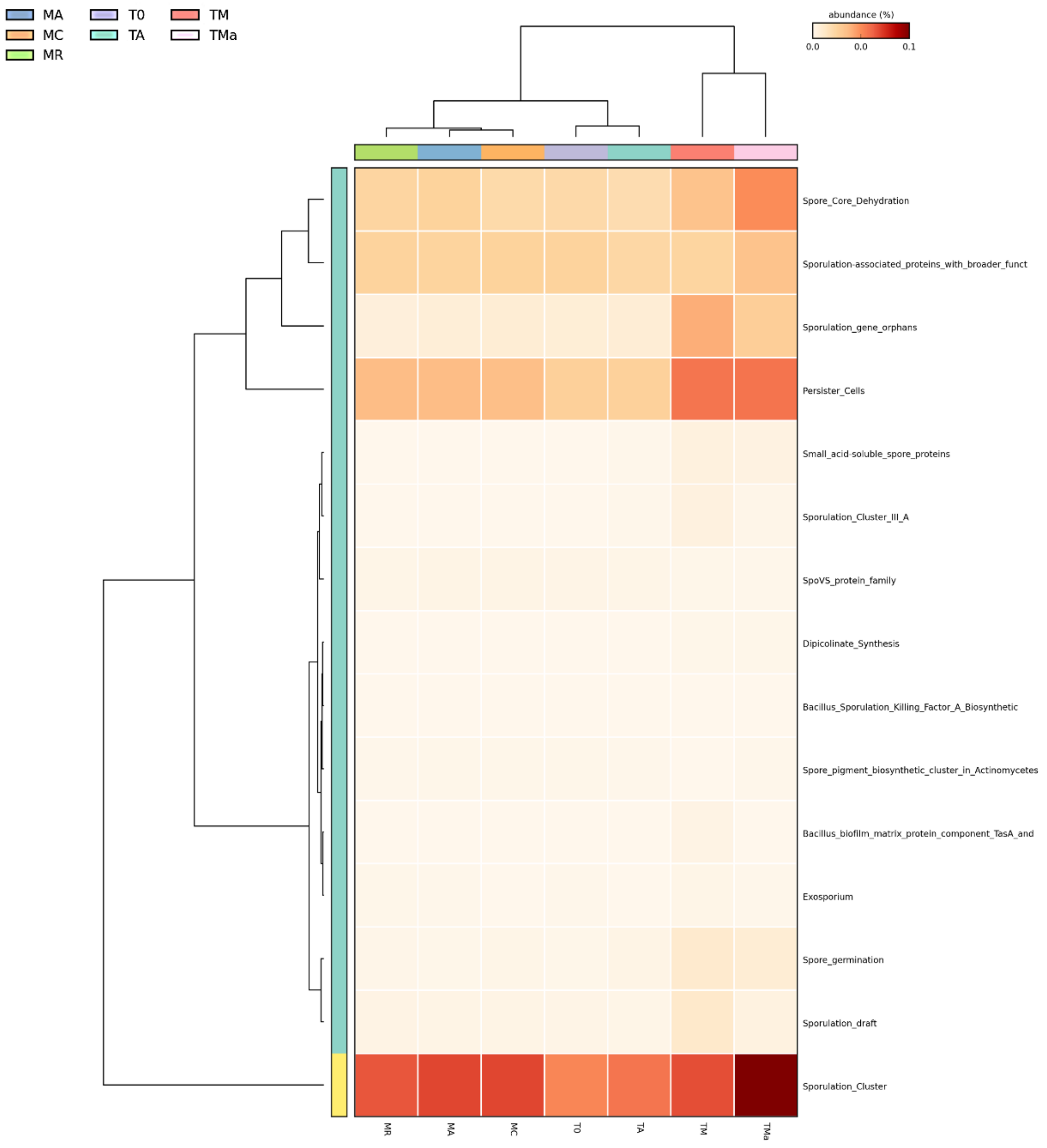

Figure S17. Heat map for sporulation and dormancy level level 3 metabolism (MG-RAST) for all metagenomes analyzed. MR: sediments collected above mangrove roots. MA: sediments collected c.a. 1.5 m from mangrove roots. MC: sediments collected from running water channel with no mangrove root influence. T0: sediments from mangrove water channel (MC) sampled in 2018. TA: enrichment water control. TM and TMa: enrichment experiments without and with antibiotics (cycloheximide 10 µg/ml, gentamycin 10 µg/ml and rifampicin 1 µg/ml), respectively.

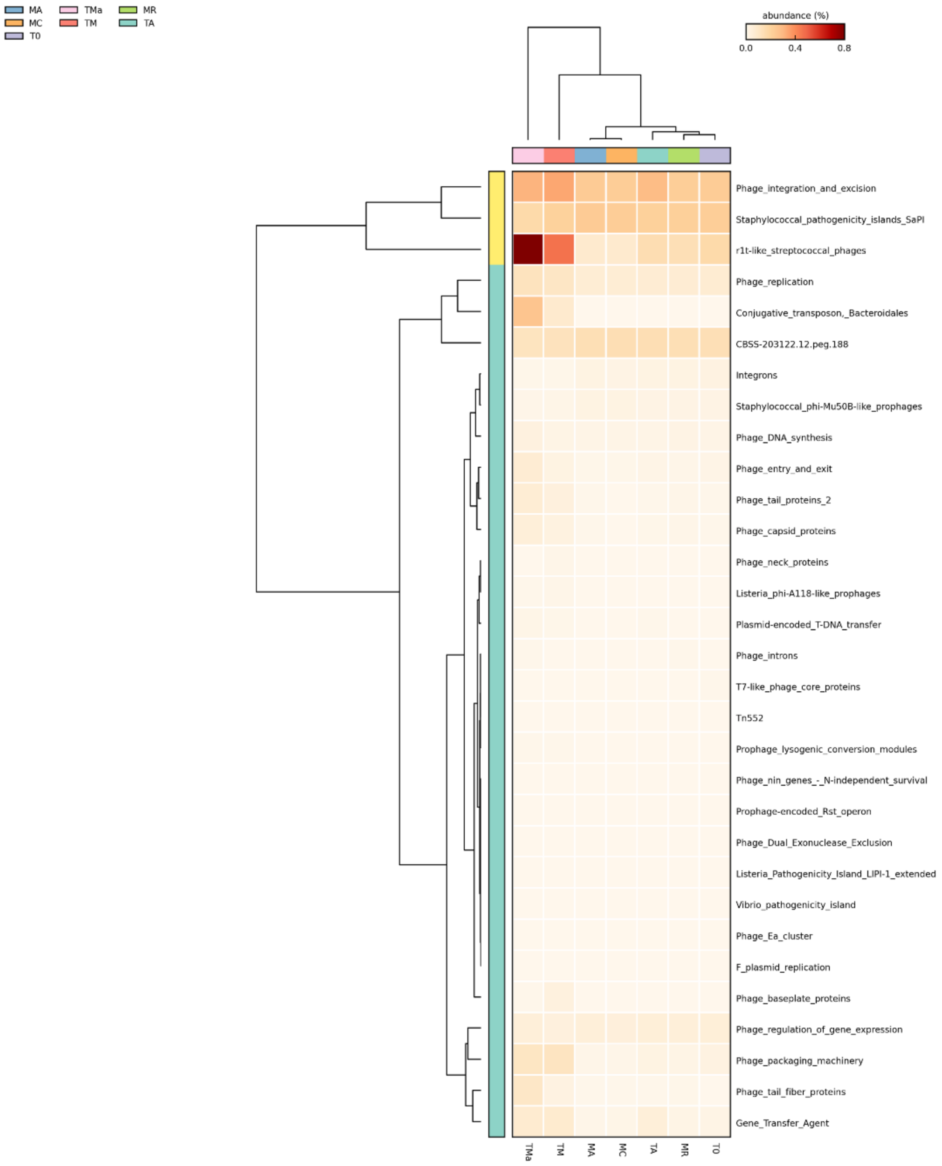

Figure S18. Heat map for phage metabolism (MG-RAST) for all metagenomes analyzed. MR: sediments collected above mangrove roots. MA: sediments collected c.a. 1.5 m from mangrove roots. MC: sediments collected from running water channel with no mangrove root influence. T0: sediments from mangrove water channel (MC) sampled in 2018. TA: enrichment water control. TM and TMa: enrichment experiments without and with antibiotics (cycloheximide 10 µg/ml, gentamycin 10 µg/ml and rifampicin 1 µg/ml), respectively.

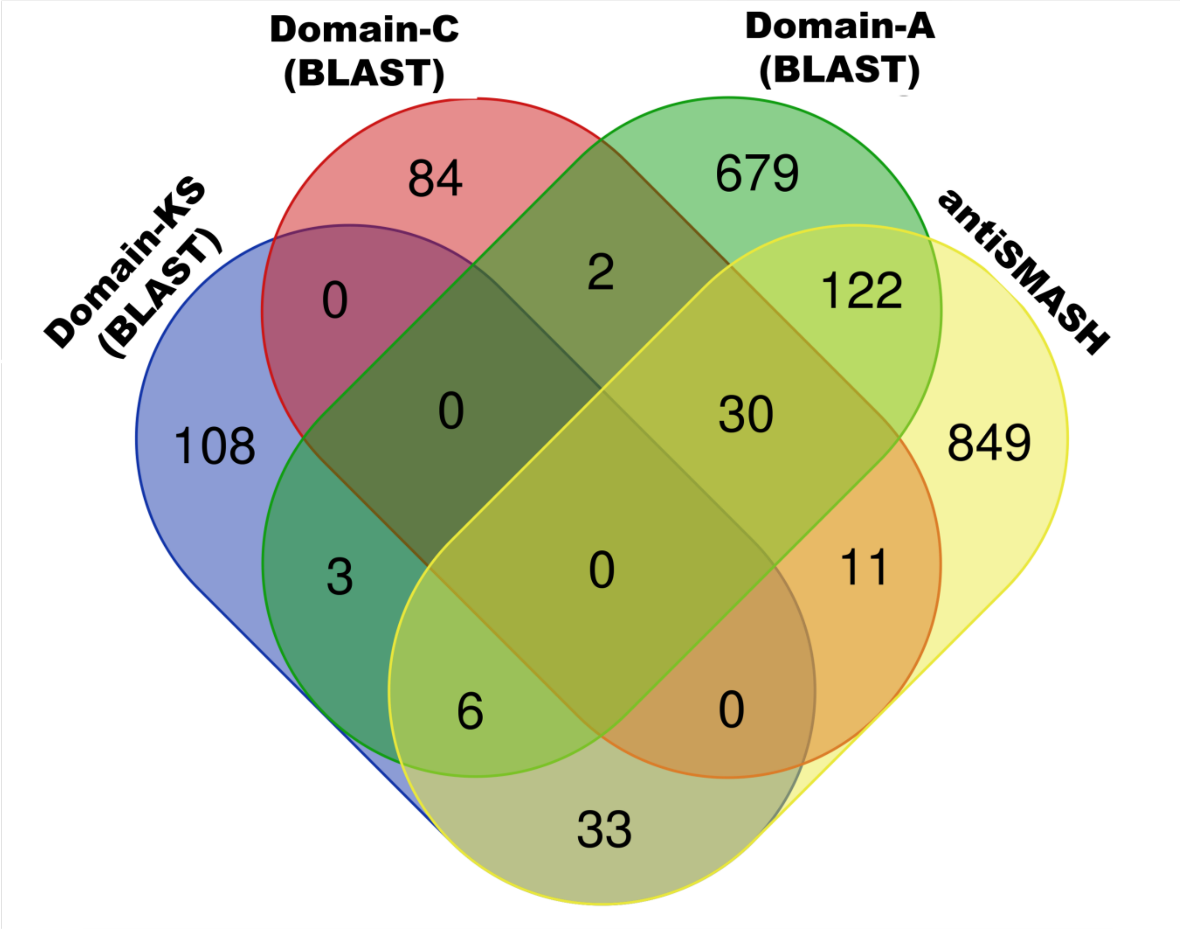

Figure S19. Venn diagram for KS and NRPS sequences found in all metagenomes analyzed using different methods.
